# Metagenomic insights into the functions of microbial communities in sulfur-rich sediment of a shallow-water hydrothermal vent off Kueishan Island

**DOI:** 10.3389/fmicb.2022.992034

**Published:** 2022-11-30

**Authors:** Li Wang, Ziyi Shen, Xinyi Cheng, Jiang-Shiou Hwang, Yizhe Guo, Mingye Sun, Junwei Cao, Rulong Liu, Jiasong Fang

**Affiliations:** ^1^Shanghai Engineering Research Center of Hadal Science and Technology, College of Marine Sciences, Shanghai Ocean University, Shanghai, China; ^2^Institute of Marine Biology, National Taiwan Ocean University, Keelung, Taiwan; ^3^Laboratory for Marine Biology and Biotechnology, Qingdao National Laboratory for Marine Science and Technology, Qingdao, China

**Keywords:** Campylobacteria, shallow-water hydrothermal vent, sulfur cycle, sediment, metagenome-assembled-genomes, Kueishan Island

## Abstract

Hydrothermal vent (HTV) systems are important habitats for understanding the biological processes of extremophiles on Earth and their relative contributions to material and energy cycles in the ocean. Current understanding on hydrothermal systems have been primarily focused on deep-sea HTVs, and little is known about the functions and metabolisms of microorganisms in shallow-water HTVs (SW-HTVs), which are distinguished from deep-sea HTVs by a depth limit of 200 m. In this study, we analyzed metagenomes of sulfur-rich sediment samples collected from a SW-HTV of Kueishan Island, located in a marginal sea of the western Pacific Ocean. Comparing with a previously published report of pelagic samples from the nearby sampling site, microbial communities in the SW-HTV sediments enriching with genes of both aerobic and anaerobic respiration inferred variable environments in the tested sediments. Abundant genes of energy metabolism encoding sulfur oxidation, H_2_ oxidation, and carbon fixation were detected from the sediment samples. Sixty-eight metagenome-assembled-genomes (MAGs) were reconstructed to further understand the metabolism and potential interactions between different microbial taxa in the SW-HTVs sediment. MAGs with the highest abundant were chemolithotrophic sulfur-oxidization bacteria, including *Sulfurovum* represented Campylobacteria involved *sox* multienzyme, sulfide oxidation genes and rTCA cycle, and Gammaproteobacteria involved *dsr* gene and CBB cycle. In addition, Desulfobacterota with the potential to participate in sulfur-disproportionating processes also had higher abundance than the sample’s overall mean value. The interaction of these bacterial groups allows the microbial communities to efficiently metabolize a large variety of sulfur compounds. In addition, the potential to use simple organic carbon, such as acetate, was found in chemolithotrophic Campylobacterial MAGs. Collectively, our results revealed the complexity of environmental conditions of the vent sediment and highlight the interactive relationships of the dominant microbial populations in driving sulfur cycles in the SW-HTV sediments off Kueishan Island.

## Introduction

Hydrothermal vents (HTVs) are sites in the seafloor where geothermally heated water is expelled through fissures in Earth’s crust. They can be found globally along spreading zones in the mid-ocean ridges or volcanic arcs (Tarasov et al., 2005). Environmental conditions at HTVs, e.g., elevated temperature and enrichment of reduced compounds, gases and heavy metals, are significantly different from those in other marine habitats (Tarasov et al., 2005). Vigorously venting black smokers on the ocean floor can reach temperatures of >400°C at 2 ~ 8 km depth. The same process also supports more subdued, lower-temperature diffuse flow systems that typically emit fluids at 5 ~ 50°C, and white smoker vents with warmer fluids (100 ~ 300°C). Elements (such as Cu, Zn, Fe, Pb, S, and SiO_2_), and volatiles (such as CO_2_, H_2_S, H_2_, and CH_4_) are incorporated into the fluids through leaching out of the rocks and direct degassing of magma chambers, respectively (Kelley et al., 2002). Since the discovery of deep-sea HTVs (DS-HTVs) in the Pacific Ocean in 1977, they have attracted wide attention. In the deep sea, far away from the sun, chemoautotrophic microbes at the base of the ecosystem derive their carbon and energy from volatile dissolved chemicals, thereby supporting the huge biomass of larger organisms (tube worms, clams, crabs etc.).The unexpectedly abundant microbial inhabitants in HTVs’ ecosystems have refined our understanding of extremophiles on Earth and their relative contribution to material and energy cycles.

Shallow-water HTVs (SW-HTVs), another type of vent system locating at depth of <200 m, are more accessible than DS-HTVs. The depth limit of 200 m is set to distinguish between “deep-sea” and “shallow-water” HTVs (Tarasov et al., 2005), coincides with the average depth of the photic zone and with a large change in the slope of the seawater boiling curve. Characteristics such as the types of gas discharges, iron or sulfur component of subsurface, and temperature changed among vents (Price and Giovannelli, 2017), but there are some common traits for SW-HTVs. As the lower pressures in shallow depth, free gas phases present in SW-HTVs, which was called “gasohydrothermal” vents in the past. Enhanced gas exsolution in the form of free gas bubbles or degassing is substantially altered fluid chemistry by mass transfer from gas to aqueous phase. Additionally, due to their shallow nature and proximity to land masses, a significant amount of terrigenous organic carbon and primary production is in direct contact with vent processes. Also, low salinity or nearly salt-free vent fluids derived from the meteoric water can be found off Ambitle Island, Papua New Guinea and Dominica Island (Lesser Antilles). As a result, the presence of light, terrestrial inputs, tidal cycles, and meteoric water inputs give rise to highly diverse and complex microbial communities in SW-HTVs (Tarasov et al., 2005; Price and Giovannelli, 2017). However, the nature of such interactions has only recently begun to be investigated, and much less studies of microbial community have been reported at SW-HTV than its deep-sea cousin.

Kueishan Island (also known as Guishan Island) is a young volcanic island located to the southernmost part of the Okinawa Trough of the western Pacific Ocean (Liu, 1995). A cluster of more than 30 type I and II vents (Giovannelli and Price, 2019) spewing sulfur-rich plumes with temperature up to 112°C and pH of 1.9–4.6 are located at depths between 10 and 80 m off the southeastern tip of Kueishan Island, while more than half of these vents are at depths <20 m (Jeng et al., 2004; Chen et al., 2005b). The hydrothermal fluid and volcanic gases contain high concentrations of CO_2_ and H_2_S (Yang et al., 2005). Wide distribution of sulfur chimney, sulfur sand, sulfur ball was observed in the seabed (Zeng et al., 2011). The yellow sulfur chimneys are composed of nearly pure sulfur (>99%; Chen et al., 2005a; Zeng et al., 2007) while more than 97% of the native sulfur is present in the form of sulfur balls (Zeng et al., 2011). Comparison with samples out of the vent area, seldom photosynthetic Chromatiales order (Gammaproteobacteria) and eukaryotic were detected in the sediment sample in Kueishan Island area by 16S rRNA gene (Wang et al., 2015). However, bacterial genera *Nautilia* and *Sulfurovum* of class Campylobacteria were reported as the dominant bacteria in low-temperature vent fluids and sediments, respectively (Zhang et al., 2012; Wang et al., 2015, 2017). Chemolithotrophic bacteria of the class Campylobacteria (formerly Epsilonproteobacteria; reclassified as a class of phylum Campylobacterota; Waite et al., 2017) have been found to be one of the most important taxa in diverse biomes of DS-HTVs (Campbell et al., 2006; Dick, 2019) and sulfur-rich shallow-water vents (Price and Giovannelli, 2017). Existing studies consistently indicated Campylobacteria occupying a high-sulfide, low-oxygen and mesophilic temperatures niche (Nakagawa and Takai, 2008; Hügler and Sievert, 2010; Meier et al., 2017; Dick, 2019). The metagenomics and metatranscriptomics studies in Kueishan Island (Tang et al., 2013; Li et al., 2018) showed that the Campylobacteria were involved in carbon fixation, sulfur oxidation, and nitrate reduction in the water column above the vents, similar to their roles in deep-sea vent systems. In order to know more special traits of these Campylobacteria thrived in SW-HTVs of Kueishan Island, we investigated for the first time the functional potentials of benthic microbial communities in the vent system off Kueishan Island by metagenomic analysis. The genes involving in energy yielding of dominant chemolithotrophic Campylobacteria and other abundant bacterial groups are analyzed based on metagenome-assembled genomes (MAGs). The potential ecological roles and possible interactions of the dominant bacterial taxa will be discussed. The study will advance the knowledge on metabolic mechanisms and ecological functions of microorganisms in SW-HTVs.

## Materials and methods

### Sample collection

Sediment cores (~10 cm) were collected from site W (121.96232°E, 24.83420°N), located 2 m away from a low-temperature vent (type II) off Kueishan Island, by scuba divers in September 2013. The sampling details and environmental parameter can be found in our previous study (Wang et al., 2015, 2017). Triplicate samples (W1, W2 and W3) were taken and immediately frozen on shipboard. In the laboratory, the samples were stored at −80°C until DNA extraction and other analyses.

### DNA extraction and metagenomic sequencing

DNA was extracted from the sediment samples after removal of the top 1 cm using the PowerSoil DNA Isolation Kit (MO BIO Laboratories, Inc., Carlsbad, United States) according to the manufacturer’s instructions. Environmental DNA of the samples was break into short fragment around 350 bp and added the adaptor. Then paired-end sequenced on an Illumina HiSeq 4,000 platform (Illumina, San Diego, CA, United States) according to standard protocols by the Beijing Genomics Institute (BGI, Shenzhen, China). Retrieved reads were filtered using an in-house developed program to remove low-quality reads by BGI. The sequencing data have been submitted to the NCBI database (accession number PRJNA851985).

### Comparative metagenomic analyses

Raw sequencing reads were submitted to the MG-RAST server (version 3.0) for gene annotation (Meyer et al., 2008).^1^ The artificially created duplicate reads (Gomez-Alvarez et al., 2009) were removed automatically by MG-RAST. The putative open reading frames (ORFs) were identified using FragGeneScen (Rho et al., 2010), and their corresponding protein sequences were searched with BLAST against the M5NR non-redundant protein database (Wilke et al., 2012) in the MG-RAST. M5NR is an integration of many databases, including the NCBI GenBank, COG, Kyoto Encyclopedia of Genes and Genomes (KEGG), and SEED. Taxonomic and functional profiles within MG-RAST (hits to IMG, M5NRA, SEED, COG, and KEGG databases) were extracted (an E-value cutoff of less than 1 × 10^−5^, a minimum read length of 50 bp and a minimum abundance of 100).

The functional attributes of our three sediment metagenomes from this study (MG-RAST ID: 4716389.3, 4716390.3 and 4716391.3) were compared with the two pelagic metagenomes previously published (Tang et al., 2013) that was stored in MG-RAST database (MG-RAST ID: 4487625.3 and 4487624.3) and hereby referred to as GS4 and GS7. In addition, nine datasets of DS-HTVs (Supplementary Table S1) were also chosen from MG-RAST to compare with the microbial communities in SW-HTVs (Grzymski et al., 2008; Brazelton and Baross, 2009; Anderson et al., 2014; Cerqueira et al., 2018; Namirimu et al., 2022). The files (“MG-RAST ID”0.299.screen.passed.fna) contained all the sequences which were passed the quality control steps in MG-RAST were downloaded to calculate the average coverage for all the collected metagenomic datasets using Nonpareil tool (Rodriguez-R and Konstantinidis, 2014). The taxonomical profile was also used to calculate the alpha-diversity using mothur (Schloss et al., 2009). The resulting profiles from MG-RAST were further analyzed using statistical software STAMP (Parks et al., 2014).

### Sequences processing, assembly, binning, and annotation

All raw sequencing reads from the triplicates (W1, W2 and W3) were assembled into contigs employing IDBA-UD algorithm with the optimized kmer 98 (Peng et al., 2012). Metagenomic binning was then conducted for assemblies longer than 2,500 bp using MetaBAT 2 (Kang et al., 2019). Redundant metagenome-assembled-genomes (MAGs) were subsequently dereplicated using dRep v. 2.3.2 (Olm et al., 2017) at 99% average nucleotide identity (ANI), and MAGs with the highest quality was selected from each cluster for downstream analysis. Qualities of the MAGs were assessed by CheckM v1.1.2 (Parks et al., 2015). Only MAGs with more than 60% completeness and less than 5% contamination were selected, all the qualified MAGs were imported into anvi’o v6.2 (Eren et al., 2015) to summary the genomic information of MAGs including abundance. Briefly, metagenomic reads were mapped to contigs using Bowtie2 (Langmead and Salzberg, 2012). The mapping results were converted into sorted and indexed BAM files for each samples (W1, W2 and W3) using samtools (Li et al., 2009). The result file of samtools and MAGs genomes was input to anvi’o to estimate the abundance and other genomics information for all MAGs.

### Taxonomic and functional annotation of MAGs

Taxonomy of the MAGs were assigned by multiple approaches. In addition to the taxonomic annotations reported by CheckM, detailed taxonomic classification of the MAGs was determined using GTDB-Tk (Chaumeil et al., 2019) based on the phylogenetically calibrated Genome Taxonomy Database (GTDB). For the interested phylum /class, phylophlan (Asnicar et al., 2020) were used to construct phylogenetic tree. As some MAGs cannot get their clear genus information from GTDB or CheckM, the genus level classification of each MAGs was also inferred from the phylogenetic tree. To ascribe functions of the MAGs, coding sequences predicted by Prodigal (Hyatt et al., 2010) were annotated using BlastKOALA against the KEGG database with default parameters (Kanehisa et al., 2016).

### Metagenomic fragment recruitment

The distribution of retrieved MAGs was studied *via* reads recruitment against published metagenomes from SW- and DS-HTVs environments (Supplementary Table S1). Raw reads were downloaded from NCBI-SRA or MG-RAST. The procedure of reads recruitment followed Liu et al. (2022). Briefly, rRNA gene sequences in MAGs were first masked to avoid bias in recruitment results. Recruitments were performed using BLASTN, and hits were filtered with a length cut-off of 50 bp, an identity cut-off of 95%, and an e-value cut-off of 1e^−5^. Qualified hits were used to compute the RPKG (reads recruited per kilobase of genome per gigabase of metagenome) values, which reflect a normalized abundance of MAGs allowing the comparison across different metagenomes.

## Results and discussion

### Comparative metagenomic analysis between the sediment and pelagic microbiome in the SW-HTVs

**Figure 1:**
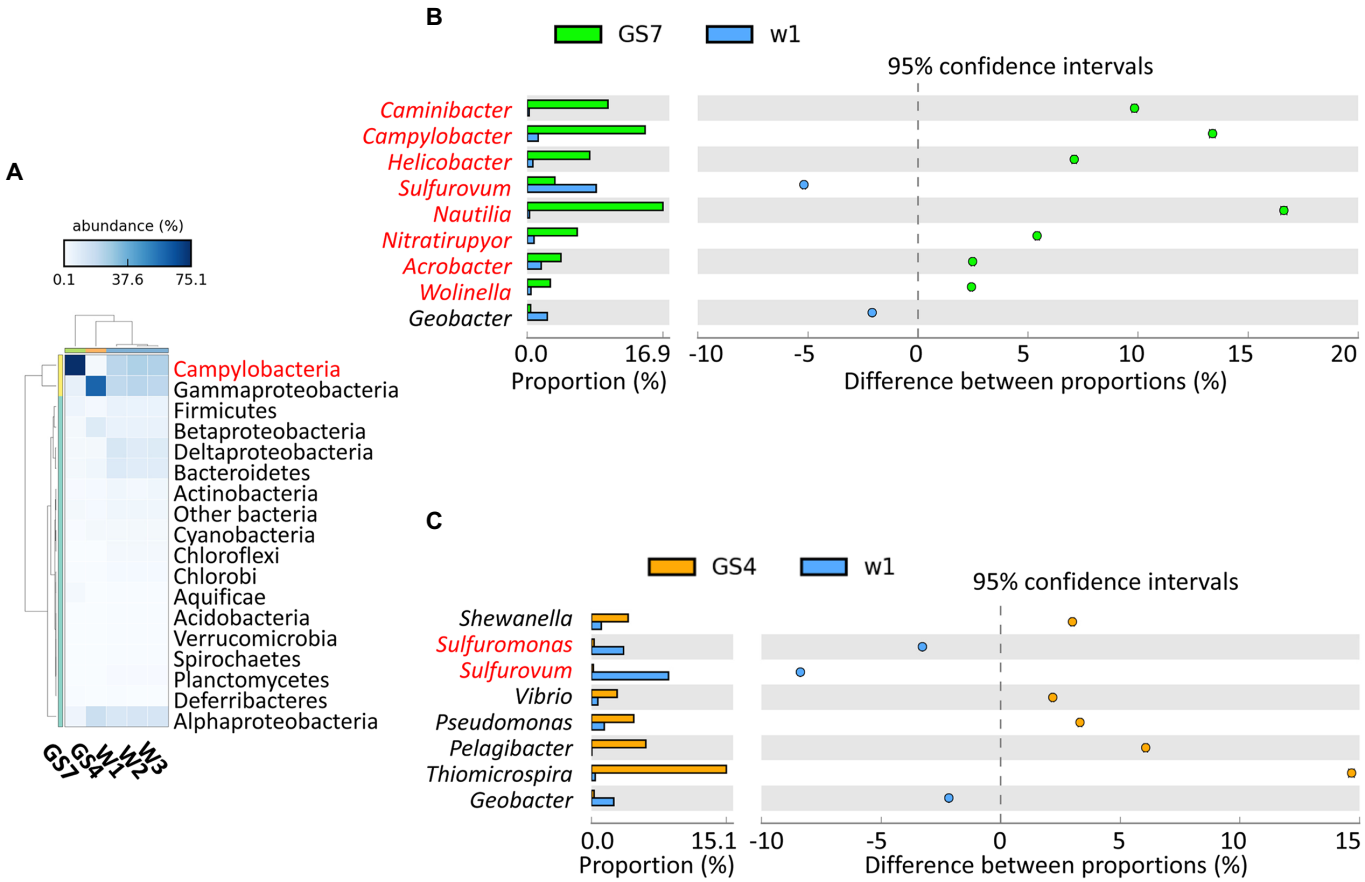
The taxonomic profile of typical phyla or classes (for Proteobacteria) **(A)** and extended error bar plot for the bacterial genera that have a difference of at least 2% between the vent fluid (GS7) or surface seawater (GS4) with sediment sample W1 **(B,C)**. “Other bacteria” in the phylogenetic classification include those with abundance<0.5% of total bacteria. The genera in red belonged to Campylobacteria.

A total of ~40 Gbp unique sequence data was generated for W1, and ~ 5 Gbp for W2 and W3. To eliminate possible impacts of sequencing depth, 5 Gb sequences of W1 was extracted up-loaded to MG-RAST together with the data of W2 and W3. In the MG-RAST pipeline, the three sediment samples (W1, W2 and W3) were analyzed and compared with two previously published metagenomes of pelagic samples, i.e., GS7 and GS4 collected from vent fluid and surface seawater above the vent, respectively (Tang et al., 2013). Taxonomic profile showed that Campylobacteria and Gammaproteobacteria were the dominant class/phylum in all three sediment samples (Figure 1A). Campylobacteria also dominated in vent fluid sample GS7 (Figure 1A), but the compositions of benthic sediment Campylobacteria were apparently different from those in the vent fluid (Figure 1B). Genus *Sulfurovum* was enriched in sediment sample W1, while genera *Nautila*, *Camylobacter* and *Caminibacter* were greatly enriched in vent fluid sample GS7 (Figure 1B). These results are consistent with findings in previous investigations by 16S rRNA gene (Zhang et al., 2012; Wang et al., 2017). It has been reported that the reduced conditions of vent fluids (high concentration of H_2_ and other reduced chemicals but out of oxygen) benefited the growth of *Nautilia* which is strictly anaerobic (Nakagawa and Takai, 2014), while the environment in the vent sediments were greatly fluctuated and might facilitate the thriving of facultative aerobic *Sulfurovum* and *Sulfurimonas* (Campbell et al., 2006; Sievert et al., 2008; Yamamoto et al., 2010). Different from the sediment and vent fluid microbiomes, the microbial communities in the surface seawater above vent (sample GS4) were dominated by *Thiomicrospira* of the Gammaproteobacteria (Figures 1A,C). Existing data showed that the genus *Thiomicrospira* mainly contained aerobic, sulfur-oxidizing and mesophilic species, found in hydrothermal environments and sulfide-rich sediment–water interfaces worldwide (Teske, 2009). Comparing with facultative aerobic *Sulfurovum* (Campylobacteria), the Gammaproteobacterial autotrophs (such as *Thiomicrospira*) preferred higher concentrations of dissolved oxygen (Teske, 2009; Dick, 2019). Overall, these results indicated that the composition of the microbial communities in different habitats around the Kueishan Island SW-HTV were greatly controlled by the prevailing redox environments in the vent fluid (GS7), the sediment (W) and the surface water (GS4).

**Figure 2:**
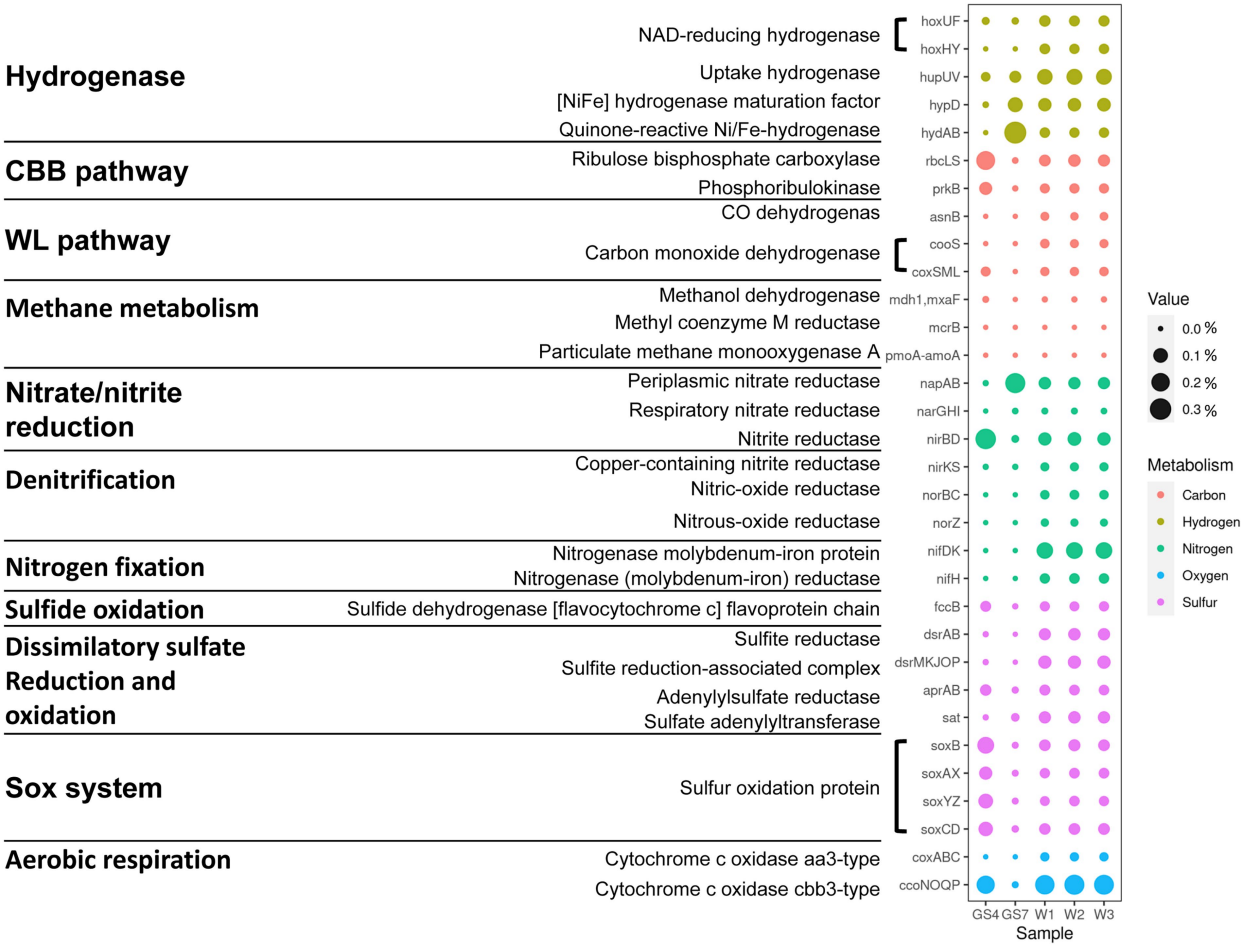
Differences in key genes involved in carbon, hydrogen, nitrogen, sulfur and oxygen metabolism among the SW-HTV sediment samples.

**Figure 3:**
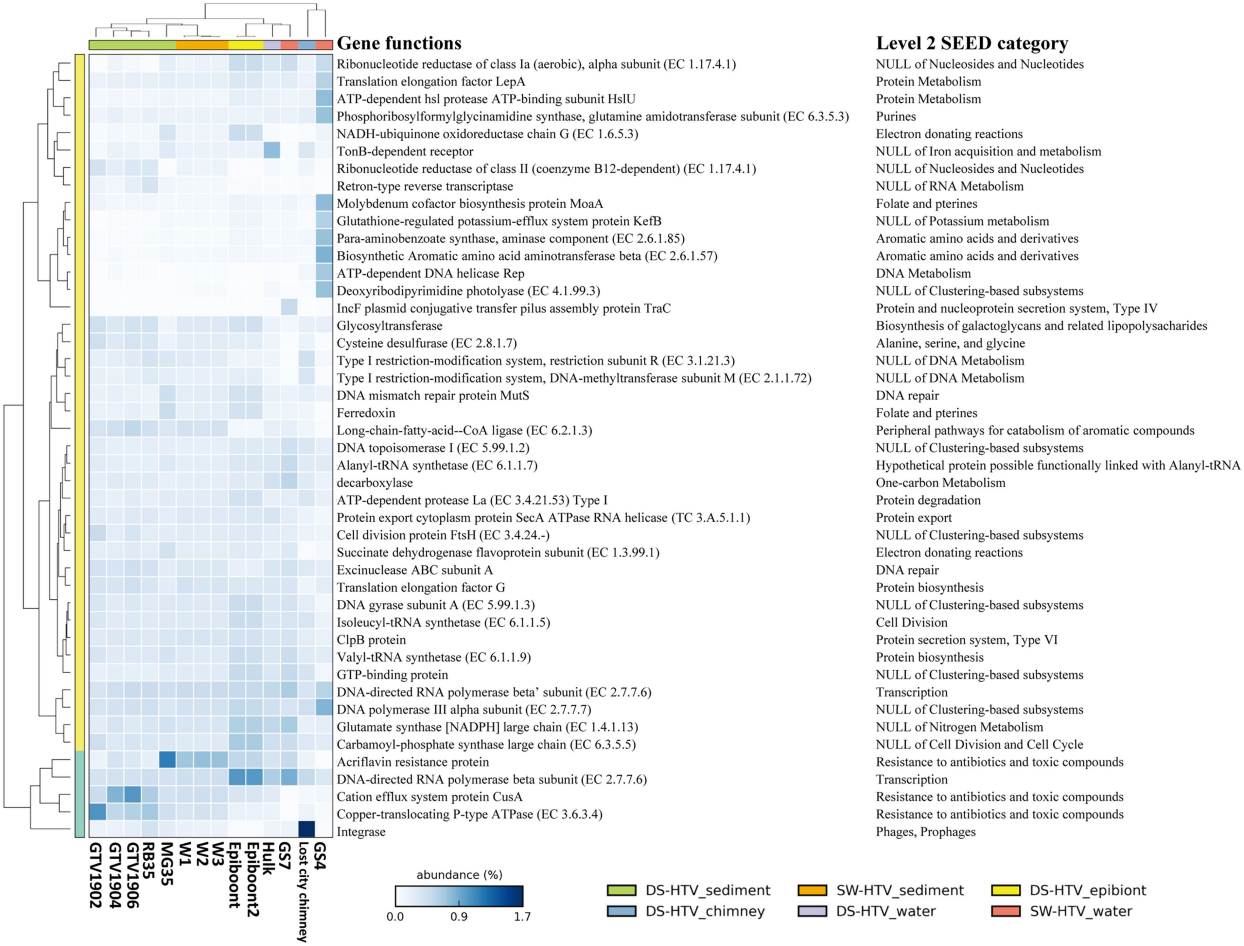
The 10 most abundant functions in our data and collected metagenomic datasets form both SW- and DS-HTVs. The dendrogram for the samples (the row) and the function (the column) were cluster by furthest neighbor and average neighbor (UPGMA) method, respectively. The name of level 2 SEED category is also listed for the functional genes.

The functional gene profiles also reflected the distinctive patterns related with oxygen availability among the three biomes. In total, about 53–57% of the identified genes were annotated for the analyzed sediment samples. Based on the annotated results of SEED subsystem, the major SEED categories (Supplementary Figures S1–S3), the key genes involving in energy metabolism (Figure 2) and the most abundant genes (Figure 3; Supplementary Figure S4) were further analyzed. Being consistent with the dominance of facultative aerobic *Sulfurovum* in the sediments (Figure 1C), the metagenomes from vent sediments showed genetic features for both anaerobic and aerobic metabolisms. On the one hand, the sediment metagenomes contained more genes related with anaerobic processes than in the surface water (GS4): the category “electron donating/accepting reaction” (level 2) was significantly distinct between the sediment sample W1 and pelagic sample GS4 (Supplementary Figure S2). Their subgroups “anaerobic respiratory reductases” (level 3) was found to be significantly higher in W1 than GS4 (Supplementary Figure S3A). Moreover, the genes related with anaerobic respiration, e.g., *napAB* which encode dissimilatory nitrate reductase and hydrogenase, were higher in sediment samples than GS4 (Figure 2). On the other hand, the sediment metagenomes contained more genes related to aerobic processes than those in the vent fluids. Diverse aerobic processes were identified from the sediment samples, including genes of Sox (sulfur-oxidation) system (*soxABCDXYZ* encoded), sulfide oxidation (*fccB* encoded), and cbb3-type O_2_ metabolism (*ccoNOQP* encoded). The relative abundances of genes involved in the above processes were much higher in sediment metagenomes than in vent fluid sample GS7 (Figure 2). In addition, the sediment metagenomes exhibited the highest ratio of genes involving in dissimilatory sulfate reduction/oxidation (such as *dsrAB*, *sat*), nitrogenase (*nifDK and nifH*) and denitrification (*nirKS* encoding nitrite reductase, *norBC* encoding nitric oxide reductase and *norZ*) among the three biomes compared (sediments, vent fluid, and surface water; Figure 2). As a result, the genes encoding both aerobic and anaerobic respiration, including O_2_/nitrate reduction, sulfur oxidation, and H_2_ oxidation, were retrieved from the sediment samples, indicating variable environments in the tested samples (W1, W2 and W3).

Genes related with “resistance to antibiotics and toxic compounds” (level 2; Supplementary Figure S2) were greatly enriched in the vent sediments than any of the pelagic samples. In the comparison of 10 most abundant genes among vents, three genes of “resistance to antibiotics and toxic compounds” (level 2) were preferentially enriched in SW- and DS-HTVs’ sediment metagenomes (Figure 3), including “cation efflux system protein *CusA*,” “copper-translocating P-type ATPase” and “acriflavine resistance protein.” The former two genes implicated the role of copper homeostasis in the sediments, while the third gene encodes a kind of multidrug resistance efflux system to protect bacteria by decreasing the intracellular concentration of acriflavine, other dyes and sodium dodecylsulfate (Nakamura, 1968). Acriflavine was used as typical antiseptic for freshwater fish as well as marine fish. The higher ratio of resistant genes of toxic compound and aquaculture drugs may indicate the accumulation of related chemicals in the sediments, which might be sourced from the hydrothermal activities (such as trace metal) or anthropogenic sources (such as aquaculture around the SW-HTVs). Fluid chemistry characteristics present both opportunities and lethal challenges to hydrothermal vent organisms. As the high temperature hydrothermal fluids were rich in heavy metal and reduced chemicals, a common characteristic of vent organisms is their ability to take advantage of the carbon and energy sources provided by hydrothermal processes while evolving mechanisms to counteract the toxic effects of high concentrations of heavy metals or short-term exposure to lethal high temperatures (Kelley et al., 2002). However, in a comparative metagenomics among mangrove, ocean, forest, grassland and agricultural soil samples, acriflavine resistance proteins were found to be highly enriched in all the samples, irrespective of ecosystem types or anthropogenic activities (Imchen et al., 2018). The exact role of “acriflavine resistance protein” in SW- and DS-HTVs still needs more studies.

Although the three studied sediment samples and two published pelagic samples may not have been taken from the exactly same vent site, the sampling sites were close to each other as they were all located in the eastern vent area of the Kueishan Island with an area of only 0.5 km^2^ (Chen et al., 2005b). Furthermore, the sampling sites were all with moderate temperature (49 ~ 30°C), low pH (4.8 ~ 5.9) and shallow depth (0 ~ 21 m; Tang et al., 2013; Wang et al., 2015). Moreover, the different vents were not isolated from each other, and the vent plumes could affect the nearby vents (Han et al., 2014). All of these facts support the validity of the metagenomic comparison conducted in this study. However, our sediment samples were taken in different years from the pelagic samples, and the possible temporal variations might cause uncertainties in the comparison. Nevertheless, our preliminary study presented the first metagenomic comparisons between sediment and pelagic biomes in SW-HTVs systems, lays a foundation for future studies.

### Genomes of the dominant taxa revealed the significance of sulfur oxidization in the SW-HTV sediments

**Figure 4:**
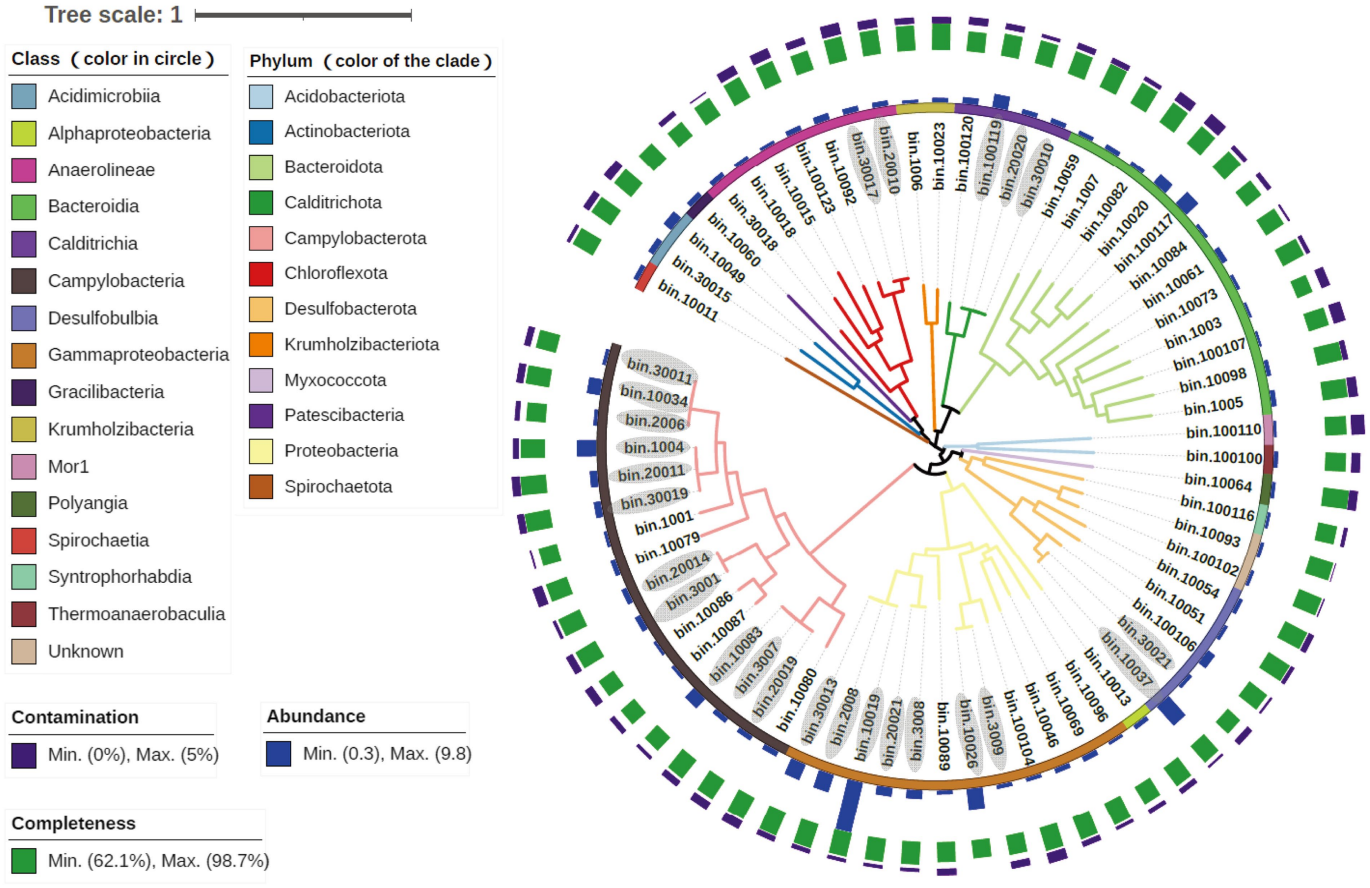
Information of all the reconstructed 69 MAGs. The colors of clades were responding to the phyla taxonomic level, while the first circle were family level. MAGs ID stating with “100” were restructed from meatgenomics of W1, “200” from W2 and “300” from W3. As the MAGs ID stands for the source of sample, their abundance referred to the ratio of that MAG’s mean coverage to that sample’s overall mean coverage. The MAGs ID with grey color own ANI value of 90 ~ 99% calculated by dRep.

To further reveal the metabolic processes and the ecological roles of the microorganisms in the SW-HTV sediments, genomes of the dominant microbial taxa were retrieved from the metagenomes *via* binning. A total of 68 MAGs (> 60% completeness and < 5% contaminations) were retrieved from all the data of three sediment samples, including. 49 from W1, 8 from W2, and 12 from W3 (Figure 4). Within them, 27 MAGs were high quality with more than 90% completeness according to the standards mentioned in Bowers et al. (2017). All the 68 MAGs were accounting for 21.68, 31.01 and 21.60% of all nucleotides in the three metagenomes, respectively. Information of the MAGs, including taxonomy, abundance, completeness, was included in Figure 4. In this study, MAG abundance was defined as its mean coverage divided by that sample’s overall mean coverage. MAGs with high abundance values were more represented in that sample than those with low abundance values. A total number of 20 “abundant MAGs” (abundance value >1) were identified, and they were belonging to phyla Desulfobulbia (bin10037 and bin100106), Campylobacteria (bin20011, bin1004, bin10034, bin30019 and bin10083), Calditrichia (bin100119 and bin100120), Bacteroidia (bin10020 and 100,117), Acidimicrobiia (bin10049 and bin30015), Chloroflexota (bin10092) and class Gammaproteobacteria (bin10019, bin10026, bin2008, bin30013, bin3008 and bin 20,021).

**Figure 5:**
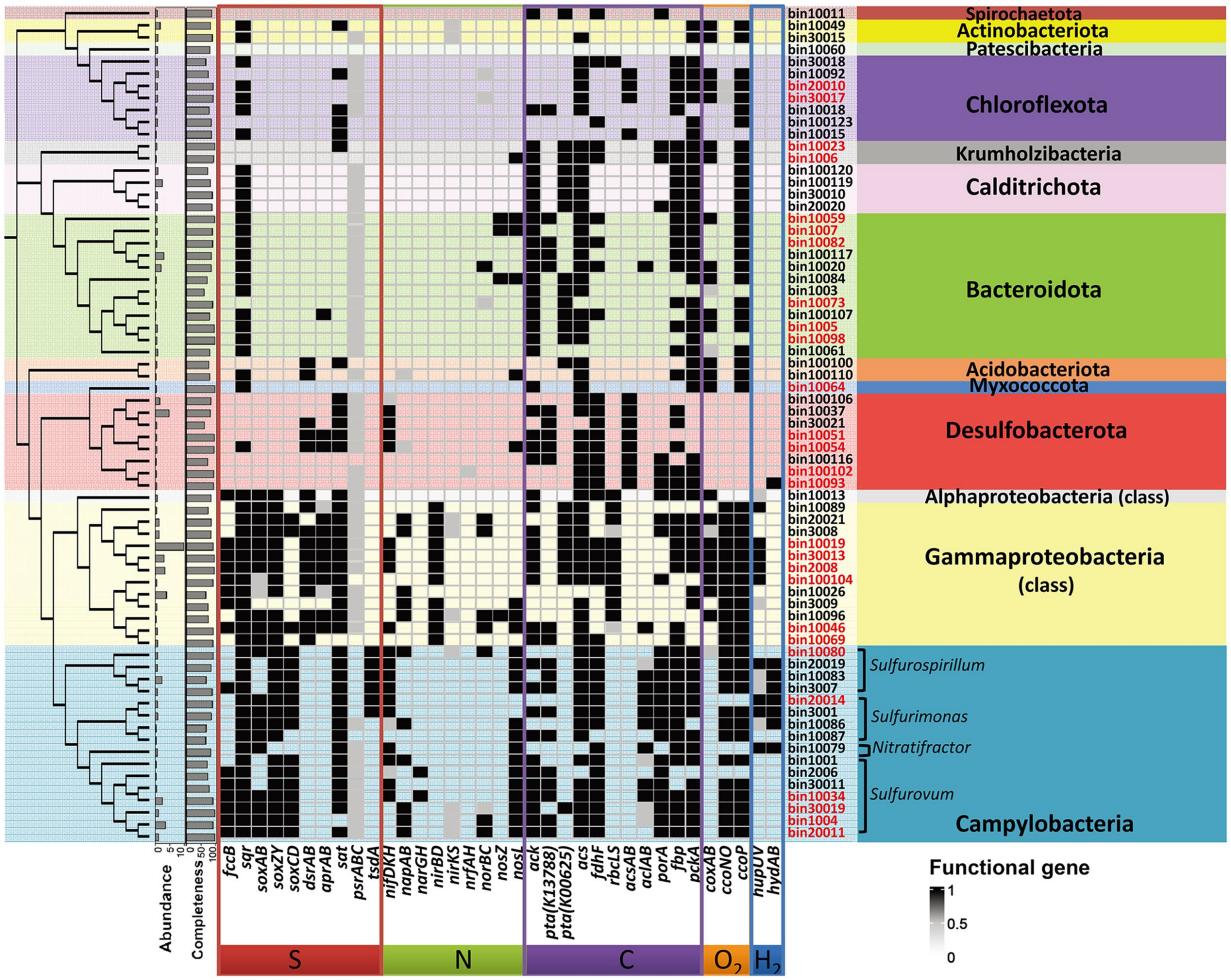
Differences in genomic information and key genes involved in carbon, hydrogen, nitrogen, sulfur and oxygen metabolism among 69 MAGs. The name of MAGs colored with red indicate the high quality MAGs with completeness >90%. The dendrogram showing the clusting of MAGs was generated from GTDB-tk. The phylum name was shown for all the MAGs. The identification of genus for the dominant Campylobacteria was inferred from the genomic phylogenetic tree (Supplementary Figure S5).

Functional annotation revealed that genes related with sulfur or sulfide oxidation processes (*soxABCDZY*, *dsrAB*, *aprAB*, *sqr* or *fccB*) were found in nearly all MAGs of Gammaproteobacteria and Campylobacteria (Figure 5), suggesting the significance of sulfur oxidization in the SW-HTV sediments. Two different modes of sulfur oxidation have been previously proposed for bacteria. The first mode involved the reverse dissimilatory sulfite reductase (rDSR, *dsrAB* encoded; Dahl et al., 2005), and the product sulfite is subsequently oxidized to sulfate by adenosine 5′-phosphosulfate (APS) reductase (*aprAB* encoded) with sulfate adenylyltransferase (*sat* encoded), or sulfite:acceptor oxidoreductase (*sor* encoded; Grimm et al., 2010). The second mode was the sulfur oxidation *via* the Sox system, which involves seven *sox* structural genes coding for four periplasmic proteins, SoxXA, SoxYZ, SoxB, and SoxCD, responsible for thiosulfate-, sulfite-, sulfur-, and hydrogen sulfide-dependent cytochrome c reduction (Friedrich et al., 2001, 2005). By these metabolic features, the sulfur-oxidizing bacteria (SOB) can oxidize reduced sulfur compounds (such as sulfide, sulfur, thiosulfide) to intermediate sulfur species (sulfite and thiosulfate) or completely to sulfate.

In our study, the 16 Campylobacterial MAGs were classified into five genera (*Sulfurovum*, *Sulfurimonas*, *Sulfuricurvum*, *Sulfurospirillum*, and *Nitratifractor*; Supplementary Figure S5). All the retrieved Campylobacterial MAGs harbored or partially harbored genes encoding the Sox system (*soxABCDZY*) but not the reverse dissimilatory sulfite reductase (rDSR, *dsrAB* gene), and the bacteria suggesting their potential ability to fix CO_2_ through rTCA cylcle (*aclB* and *porA* genes; Figure 5). Genes encoding fructose-1,6-bisphosphatase and phosphoenolpyruvate carboxykinase for gluconeogenesis were also annotated in these MAGs (Supplementary Table S2), suggesting that the fixed carbon can be stored as glucose. Similar processes have been reported in previous studies on deep-sea lineages of Campylobacteria (Waite et al., 2017). Among the Campylobacterial lineages, the genus *Sulfurovum* was identified as the most dominant taxa, reflected by either the taxonomic profile (Figure 1) or MAG abundance (Figure 4; Supplementary Table S3). Although gene *sqr* was contained in all Campylobacterial MAGs, only MAGs of the *Sulfurovum* (bin20011, bin1004, bin30019, bin10034, bin30011, bin2006, and bin1001, Figure 5) were found to contain the *fcc* gene (Figure 5). Both the membrane-bound Sqr and periplasmic Fcc enzymes catalyzed sulfide oxidation to form S^0^. However, Fcc was sensitive for the H_2_S concentration since its down-stream cytochrome c oxidase (Cco) enzyme was sensitive to HS^−^ even at moderate sulfide concentrations (Collman et al., 2009). On the contrary, Sqr was not subjected to sulfide inhibition because it used the non-cytochrome system – the quinone pool for electron transfer (Brune, 1989). The presence of both Fcc and Sqr in *Sulfurovum* enhanced their ability to use sulfide, especially under the conditions of low H_2_S concentration and the presence of O_2_. Such metabolic features would provide survival advantages for *Sulfurovum* species in the variable environmental conditions of the vent sediments.

**Figure 6:**
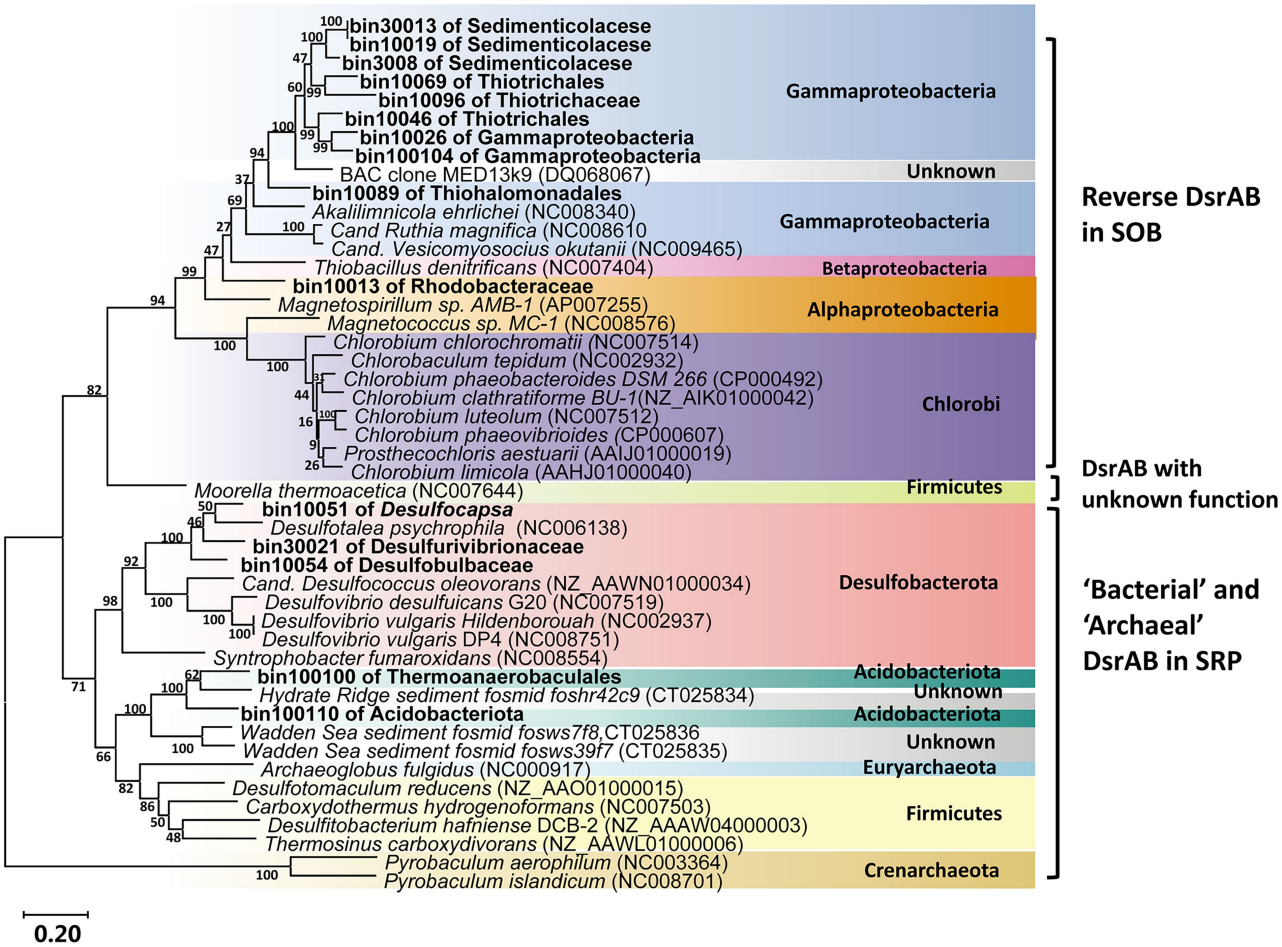
Phylogenetic tree of dsrAB amino acid sequences from all related MAGs. The reference gene was from Loy et al. (2009). Scale bar indicates 20% sequences divergence as estimated from distance-matrix analysis. The labelled names of MAGs were the lowest taxonomic level assigned by GTDB (Supplementary Table S3).

Most of the retrieved Gammaproteobacterial MAGs harbored the genes encoding incomplete Sox system lacking of *soxCD* (encoding the sulfur dehydrogenase). These Gammaproteobacterial MAGs also contained gene sets encoding the rDsr pathway, an additional pathway for sulfur oxidation. Moreover, the Gammaproteobacteria in the vent sediments showed the potential for CO_2_ fixation *via* the CBB pathway (*rbcLS*; Figure 5). These genomic features were consistent with previous reports of chemotrophic and phototrophic sulfur-oxidizing Gammaproteobacteria (Yamamoto and Takai, 2011). The rDsr enzyme was homologous to, but phylogenetically clearly distinguishable from the dissimilatory sulfite reductase catalysing the energy-conserving reduction of sulfite to sulfide in sulfite/sulfate-reducing prokaryotes (SRP). The paralogous genes *dsrA* and *dsrB*, encoding its α- and β-subunits respectively, were used as phylogenetic markers for SOB capable of sulfur storage (intracellular sulfur globules). In the phylogenetic tree of *dsrAB* gene, Gammaproteobacterial MAGs formed a highly supported monophyletic branch with other SOB, separating from typical sulfite/sulfate-reducing prokaryotes (SRP; Figure 6). Among the other retrieved MAGs, those from the phyla Desulfobacterota (bin10051, bin10054 and bin30021) and Acidobacteria (bin100100 and bin100110; Figure 6) also harbored the *dsrAB* genes (Figure 5), but they clustered with the SRP in the phylogenetic tree (Figure 6), indicating their role in sulfate reduction not sulfur oxidation. Obviously higher abundance of Gammaproteobacterial SOB containing rDsr pathway (as high as 9.81, Supplementary Table S3) than MAGs of SRP (abundance lower than the mean of community at value <0.6; Supplementary Table S3) suggested the predominance of sulfur oxidation process in the vents’ sediments of Kueishan Island.

### The potential to use simple organic carbon in chemolithotrophic campylobacteria

To date, nearly all of the existing Campylobacterial isolates from deep-sea hydrothermal vents have been described as chemolithoautotrophs (Campbell et al., 2006), fixing carbon *via* the rTCA cycle (Hügler et al., 2005). Except dissimilatory oxidizing inorganic sulfur compounds (sulfide, sulfur or thiosulfate), they gain energy by oxidizing hydrogen/formate (Waite et al., 2017) using hydrogenase and formate dehydrogenase (*fdhF* encoded). Some other simple organic substances such as acetate were also reported to be used in host-associated isolates of Campylobacteria (Campbell et al., 2006), or from the genomes of MAGs in sulfidic hydrocarbon-rich aquifer (Keller et al., 2015), and co-culture Campylobacterial groups of benzene-degrading microbial community (Starke et al., 2016), but were seldom reported in the free living microbes of vent area.

Our result showed for the first time that the Campylobacteria in SW-HTV sediments may have the potential to use acetate and some simple organic carbon, as genes related with the metabolism of acetate and some C4 organic carbon was found in the genomes of our Campylobacterial MAGs (Figure 5; Supplementary Table S2). Firstly, acetate may be absorbed and used by the cells. Acetate kinase (Ack, EC 2.7.2.1, encoded by *ack* gene) and phosphate acetyltransferase (Pta, EC 2.3.1.8, encoded by *pta* gene) presented in nearly all MAGs of *Sulfurovum* and most of other Campylobacteria MAGs (Figure 5). The Ack-Pta pathway not only catalyzes the conversion of acetyl-CoA and ADP to acetate and ATP in most facultative and strictly anaerobic microbes, but also reversibly catalyzes the activation of acetate to acetyl-CoA as a first step in the assimilation or dissimilation of acetate in some aerobic and anaerobic bacteria (Preston et al., 1989). The Ack-Pta pathway was able to dissimilate high concentrations of acetate (Kumari et al., 1995), and it also allowed the bacteria to switch from acetate production to consumption rapidly. The presence of *actP* gene encoding acetate permease for acetate absorbing and enhancing the possibility of using extracellular acetate in Campylobacteria, was found in the nearly complete MAGs bin30019 (completeness = 98%) of *Sulfurovum* (data not shown). Secondly, genes related with C4-dicarboxylates sensing and sugar transport was annotated from the MAGs of Campylobacteria. In our study, *dctB* and *dctD* were detected in Campylobacterota-like MAGs (Supplementary Table S2), which were the key genes encoding the C4-dicarboxylate transport system (Dct), a regulatory system to monitor the external concentration of C4-dicarboxylic molecules (Janausch et al., 2002). C4-dicarboxylates (fumarate, succinate, malate, oxaloacetate, aspartate, etc.) were intermediates of central metabolism in most living organisms, serving as important carbon and energy sources for growth. Besides, fumarate also can be used as electron acceptor to performed oxidative phosphorylation with H_2_ or formate as electron donors, as revealed from model organism *Wolinella succinogenes* of phylum Campylobacterota (Kröger et al., 2002). Given the great importance of the C4 dicarboxylates, the presence of Dct system would undoubtably benefit the survival of Campylobacteria in nutrient-limited environments. In addition, genes encoding multiple sugar transport system ATP-binding protein (K10112) and phospholipid/cholesterol/gamma-HCH transport system *mlaDEF* (K02065, K02066 and K02067) were annotated from genomes of genus *Nitratrifractor* of Campylobacterota (Supplementary Table S2), indicating the ability to uptake of different types of organic compounds such as disaccharides and/or oligosaccharides as well as lipids. All of these results showed the potential of Campylobacteria to perform heterotrophic metabolisms. Till now, nearly all of the existing Campylobacteria isolates from hydrothermal vents were chemolithotrophic (Campbell et al., 2006), and the mechanisms and ecological implications of their potentials for heterotrophic metabolism in SW-HTV sediments may need to be further explored in future studies.

### Complex relationships among diverse bacteria involving in sulfur metabolism

**Figure 7:**
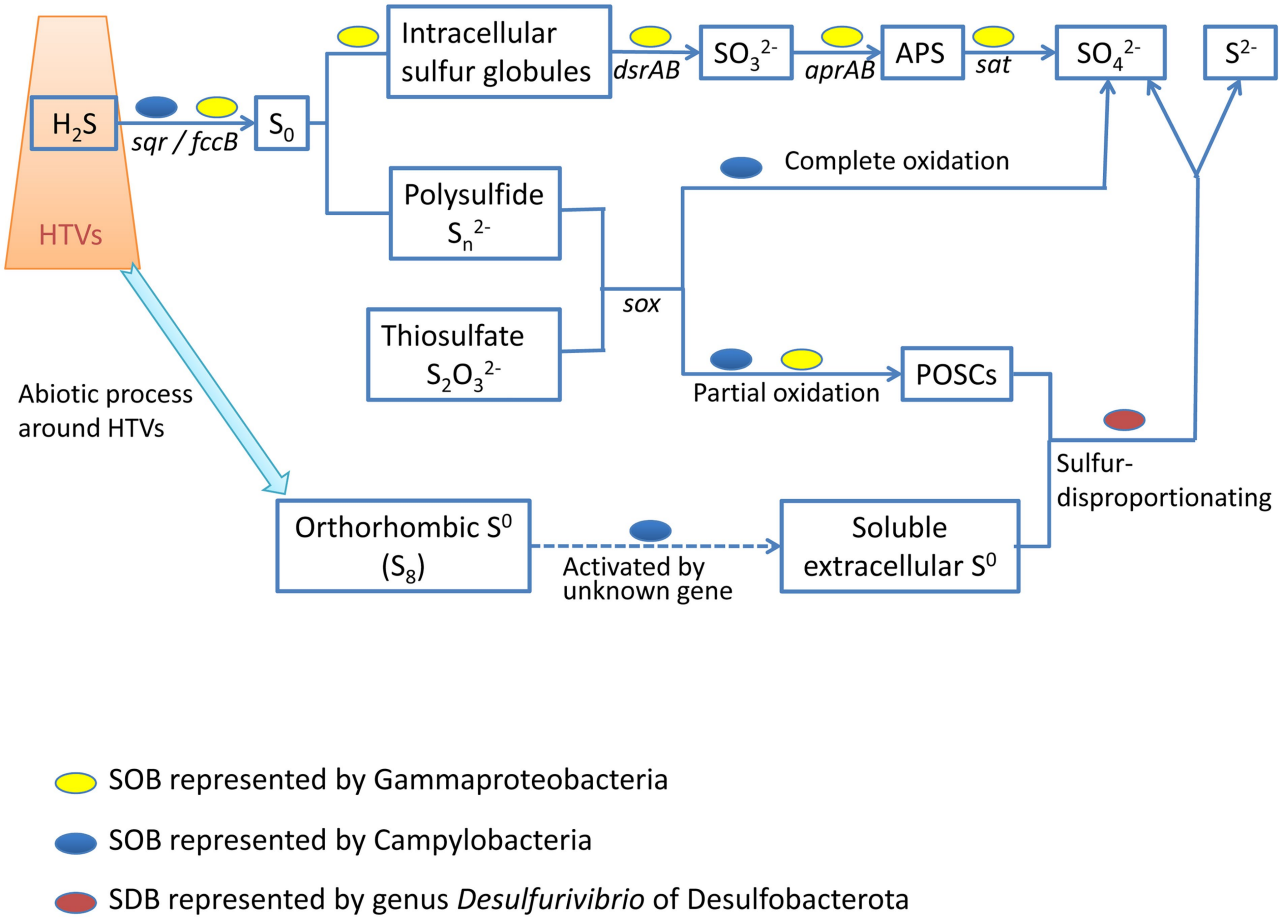
Schematic diagram showing relationship of the abundant MAGs involving in the sulfur cycle. Although both Gammaproteobacterial and Campylobacterial SOB can oxidize various sulfur form (H^2^S, Sn^2-^, S2O3^2-^, etc.), their mechanism and processes were different. Gammproteobacteria (colored with yellow) can form intracellular sulfur globules and use reverse dissimilatory sulfite reductase (encoded by dsrAB gene) to form oxidative sulfate, while Campylobacteria (colored with blue) use the Sox multi-enzymes to form sulfate, or partially oxidized sulfur compounds (POSCs) when the availability of electron acceptor such as oxygen and nitrate was limited. The formed POSCs or soluble extracellular S^0^ activated by some kind of Campylobacteria can offer growth for SDB (colored with red). SOB: sulfur-oxidizing bacteria; SDB: sulfur disproportionation bacteria.

The SW-HTV sediments we analyzed in this study were rich in sulfur with visible sulfur globules, as seen in our previous study (Wang et al., 2017). Elemental sulfur is widely distributed in the vent sediments of Kueishan island, represented by the extensive distribution of sulfur chimney, sulfur sand etc. (Zeng et al., 2011). However, the natural orthorhombic sulfur (formed abiotically, most in the form of S_8_) was hard to be directly used by microbes due to their hydrophobic nature and low aqueous solubility. In contrast, the biologically produced elemental sulfur is hydrophilic and was recognized as the “microbiologically preferred form” (Prange, 2008). For example, chainlike type R-S_n_-R with R being H, Cl, Br, SO_3_^−^ and/or organic group were usually found in phototrophs (Kleinjan et al., 2003). For chemotrophs, soluble extracellular S^0^ coating with organic envelopes rich in amide and carboxylic groups were also found in *Sulfuricurvum kujiense* (Campylobacteria; Cron et al., 2021). In our study, potential SOBs, i.e., Campylobacteria and Gammaproteobacteria were found to be the dominant bacterial taxa in the vent sediments analyzed, and their genomes harbored multiple types of energy-yielding pathways (Figure 5) allowing the oxidation of different forms of sulfur compounds, with different end products, depending on the redox conditions in the surrounding environments (Figure 7). Particularly, the bio-active element sulfur can be formed under incomplete oxidation of sulfide when the availability of electron acceptor such as oxygen and nitrate is limited, while complete sulfide oxidation to sulphate may be occurred where oxygen or nitrate is sufficient (Lavik et al., 2009).

Except the potential sulfur oxidizing Campylobacteria and Gammaproteobacteria, MAGs of phylum Desulfobacterota were also retrieved in high abundance, such as bin100106 (abundance 1.54, completeness 85.25%) and bin10037 (abundance 4.76, completeness 83.04%; Supplementary Figure S3; Supplementary Table S3). These abundant MAGs were affiliated with genus *Desulfurivibrio* of family Desulfurivibrionaceae (Supplementary Figure S6). Previous study showed that bacteria from genus *Desulfurivibrio* were incapable of driving sulfate or sulfite reduction, but can perform sulfur disproportionation utilizing sulfur compounds as electron acceptor and short-chain fatty acids or hydrogen as electron donors (Sorokin et al., 2008). The high abundance of the sulfur disproportionation bacteria (SDB) affiliated MAGs indicated sufficient substrate for their growth. As the natural orthorhombic sulfur own low aqueous solubility, SDB can use the intermediate products (biotic sulfur) formed by SOB under partial oxidization when the electron acceptor such as oxygen and nitrate is limited (Figure 7). It was also reported that chemoautotrophic strain *Sulfurimonas denitrificans* (Campylobacteria) was able to access cyclooctasulfur (S_8_) with a metabolic feature not yet demonstrated (Pjevac et al., 2014). As a result, SDB may also benefit from Campylobacteria during its activating orthorhombic sulfur (Figure 7).

Based on the physical reports of phototrophic SOB, the complete Sox pathway results in sulfate as the sole end product, whereas the incomplete pathway (with SoxCD absent) results in either intra- (Sakurai et al., 2010) or extracellular (Gregersen et al., 2011) accumulation of S^0^. However, it was found that Sox system gene clusters split into two major clusters in chemoautotrophic Campylobacteria (Nakagawa et al., 2007). Enzymatic activity measurements and partial protein purification indicated that the Sox enzyme system was constitutively expressed when H_2_ or thiosulfate as the electron donor (Yamamoto et al., 2010). As most knowledge of sulfur oxidizing process were originated and reviewed from the studies on phototrophic SOB (Frigaard and Dahl, 2008), further study on the process of sulfur oxidation need to be noticed for chemolithotrophic SOB. The detection of sulfur oxidation process under diver electron donor (H_2_, S^0^, etc.) with different concentration of electron donors should give deeper studies by transcriptomics or proteomics. Furthermore, the lack of pure cultures of the dominant sulfur oxidizers in deep-sea and shallow-water vents limited the understanding of the mechanisms. As a result, isolate SOB from vent system is needed as the first step.

### Comparative metagenomics analysis between DS-HTVs and SW-HEVs

We compared our dataset to a collection of metagenomes derived from the sediments, epibiont, water and chimney biofilm of DS-HTVs (Supplementary Table S1). As shown by the Nonpareil curve (Supplementary Figure S7), the coverage of our samples was as high as 92% when the sequences number reached 50 Gb (Supplementary Figure S7; Supplementary Table S4). Majority of the DS-HTVs sediment samples (except MG35 sediment sample) had higher Nonpareil diversity than the SW-HTVs sediments analyzed in this study (Supplementary Figure S7; Supplementary Table S4), while MG35 had comparable diversity value with ours. The sediment samples of both DS- and SW-HTVs had Shannon diversity above 5.28 except MG35, while the epibionts and GS7 had lower Shannon index (below 3.72; Supplementary Figure S8). Bacteria dominated in all of the compared metagenomes (Supplementary Table S1). Camplylobacteria was the most abundant bacterial phylum in most datasets, with higher relative abundance in GS7 of SW-HTVs, epibiont samples and sediment sample MG35 of DS-HTVs, following by the water sample from “Hulk” DS-HTVs and sediment samples from SW-HTVs (Supplementary Figure S9A). At the genus level, *Sulfurovum* was the most abundant Camplylobacteria group in SW-HTV sediment samples, whose relative abundance was comparable with the epibiont samples, but lower than sediment sample MG35 in DS-HTVs (Supplementary Figures S9B, S10). The dominance of *Sulfurovum* in MG35, W1, W2, W3 and epibiont samples (Supplementary Figure S10B) of oxic mixing zone, and with high rate of H_2_S and CO_2_ indicate the special niche for these bacteria (Cerqueira et al., 2018). For the reconstructed MAGs, their distribution among the collected datasets was shown in Supplementary Figure S11. Most of the MAGs had much higher RPKG value in the SW-HTV sediment samples analyzed in this study, suggesting an endemic distribution of the dominant bacterial species. The heatmap of top 10 abundant functions shown the acriflavin resistance protein and copper homeostasis proteins (including CusA and Copper tanslocating P-type ATPase) were abundant in the sediments of both DS- and SW-HTVs (Figure 3). The resistance of acriflavin was highest in W1, W2 W3 and MG35. The copper homeostasis proteins were higher in GTV1902, GTV1904, GTV1906 and RB35 of DS-HTVs sediments biome than the SW-HTVs, but MG35 of DS-HTVs sediments own comparable ratio with SW-HTVs sediments.

## Conclusion

In this study, we report for the first time the sediment microbial community functional potential of a shallow water hydrothermal vent off Kueishan Island. Comparative metagenomic analysis with two published pelagic datasets of the vent revealed the different microbial taxa and functional potentials of biomes in the sediments and the water column above the vent. The microbial community structure was largely controlled by the prevailing redox conditions, with the dominance of facultative aerobic *Sulfurovum* (Camplylobacteria) in the sediments, as oppose to strictly anaerobic *Nautilia* in the vent fluid and aerobic *Thiomicrospira* in seawater above the vent. Chemolithotrophs, implicated in carbon fixation, dissimilatory sulfur oxidation/reducing, dissimilatory nitrate reduction, and hydrogen oxidation were detected in SW-HTVs as their deep-sea cousin. In addition, the dominant MAGs of sulfur-disproportionation bacteria illustrated the diverse energy-yielding processes related with element sulfur in SW-HTVs. As element sulfur was the most important precipitate from fluids in SH-HTVs of Kueishan Island, the special characteristics to use element sulfur, or interactive cooperation with others to do so, may be an advantage to survive for the microbes there. The details of the sulfur related process could be followed in the future. Furthermore, we also detected the potential to use simple organic carbon from the genomes of chemolithotrophic Camplylobacterial MAGs.

## Data availability statement

The data presented in the study are deposited in the NCBI repository, accession number PRJNA851985.

## Author contributions

LW, RL, and JF designed the expedition and sampling scheme. LW, XC, and MS conducted experimental procedures including DNA extraction and PCR amplification. LW, ZS, and YG performed bioinformatical analyses including sequence processing, metagenomic comparison, binning, and phylogenetic analysis. LW and RL analyzed and summarized the data and wrote the article. J-SH managed the sampling. JC made comments and suggestions to the text. All authors contributed to the article and approved the submitted version.

## Funding

This work was supported by the National Natural Science Foundation of China (grant nos. 41906134 and 91951210), the special developmental project of Shanghai Ocean University (grant no. A2-2006–00-200217), and a direct grant for research from the research committee of The Chinese University of Hong Kong.

## Conflict of interest

The authors declare that the research was conducted in the absence of any commercial or financial relationships that could be construed as a potential conflict of interest.

## Publisher’s note

All claims expressed in this article are solely those of the authors and do not necessarily represent those of their affiliated organizations, or those of the publisher, the editors and the reviewers. Any product that may be evaluated in this article, or claim that may be made by its manufacturer, is not guaranteed or endorsed by the publisher.

## Footnotes

^1^ http://metagenomics.anl.gov/
